# Harold Frederic on Doctors

**Published:** 1899-01

**Authors:** 


### Harold Frederic on Doctors.

The late Harold Frederic, whose Christian science slayers were recently discharged from custody, was perhaps led to his doom through dislike for the medical profession. He was known to entertain a very unflattering opinion of physicians, and in his last novel, "Gloria Mundi," gave vent to his contempt in the following passage: "He drifted into an attack upon doctors as a class. He denounced them, root and branch, as impostors and parasites who darkened and embittered human life by fostering all the mean cowardices of small-brained people in order that they might secure a dishonest livelihood by pretending to dispel the horrors their own low tricks had conjured up."—*New York Medical Record.*

